# Improved motor performance in patients with acute stroke using the optimal individual attentional strategy

**DOI:** 10.1038/srep40592

**Published:** 2017-01-17

**Authors:** Takeshi Sakurada, Takeshi Nakajima, Mitsuya Morita, Masahiro Hirai, Eiju Watanabe

**Affiliations:** 1 Functional Brain Science Laboratory, Center for Development of Advanced Medical Technology, Jichi Medical University, 3311-1 Yakushiji, Shimotsuke, Tochigi, 329-0498, Japan; 2 Department of Neurosurgery, Jichi Medical University, 3311-1 Yakushiji, Shimotsuke, Tochigi, 329-0498, Japan; 3 Rehabilitation Center, Jichi Medical University Hospital, 3311-1 Yakushiji, Shimotsuke, Tochigi, 329-0498, Japan; 4 Department of Neurology, Jichi Medical University, 3311-1 Yakushiji, Shimotsuke, Tochigi, 329-0498, Japan

## Abstract

It is believed that motor performance improves when individuals direct attention to movement outcome (external focus, EF) rather than to body movement itself (internal focus, IF). However, our previous study found that an optimal individual attentional strategy depended on motor imagery ability. We explored whether the individual motor imagery ability in stroke patients also affected the optimal attentional strategy for motor control. Individual motor imagery ability was determined as either kinesthetic- or visual-dominant by a questionnaire in 28 patients and 28 healthy-controls. Participants then performed a visuomotor task that required tracing a trajectory under three attentional conditions: no instruction (NI), attention to hand movement (IF), or attention to cursor movement (EF). Movement error in the stroke group strongly depended on individual modality dominance of motor imagery. Patients with kinesthetic dominance showed higher motor accuracy under the IF condition but with concomitantly lower velocity. Alternatively, patients with visual dominance showed improvements in both speed and accuracy under the EF condition. These results suggest that the optimal attentional strategy for improving motor accuracy in stroke rehabilitation differs according to the individual dominance of motor imagery. Our findings may contribute to the development of tailor-made pre-assessment and rehabilitation programs optimized for individual cognitive abilities.

Cognitive factors such as attention direction^1^,^2^ and motor imagery ability^3^,^4^ can affect motor performance. Directional attention can follow two strategies when performing physical activity: internal focus (IF) and external focus (EF)^5^. In the IF strategy, attention is directed to body movement itself, whereas in the EF strategy, attention is directed to movement outcome. Previous studies have demonstrated the advantages of the EF strategy for motor performance in healthy populations^1^,^2^. The advantage of the EF strategy is explained by the constrained-action hypothesis; attempts to consciously monitor/control body movements (IF strategy) interfere with automatic motor control processes. However, the interference can be weakened by applying the EF strategy^6^. This hypothesis is also supported by empirical findings based on movement correction frequency^6^, attentional-capacity demands^7^, and electromyography during motor learning tasks^8^.

Like the attentional strategy, motor imagery can be categorized into two distinct modalities, kinesthetic and visual motor imageries^9^. Kinesthetic motor imagery requires simulating the feeling of muscle or joint sensations, while visual motor imagery involves mentally visualizing one’s body movements. These distinct motor imagery abilities vary across individuals, and the individual differences affect the acquisition of new movements^10^.

Although both attentional strategy and individual motor imagery ability can affect motor performance, the specific effects of IF and EF strategies^11^,^12^ and of individual abilities for kinesthetic and visual motor imageries^10^,^13^ were separately explored in most previous motor control studies. A direct link between the EF strategy and visual motor imagery has not been empirically established. In contrast, a previous study demonstrated a mutual interaction between the IF strategy and kinesthetic motor imagery process during a motor learning task^14^. In that study, participants were required to learn tango steps with conscious control of lower limbs (i.e., corresponding to the IF strategy). Following training, neural activity in several brain regions related to kinesthetic motor imagery, such as the inferior parietal lobule, was significantly increased during motor imagery compared with that during the pre-task session. These findings imply that attentional direction and motor imagery modalities, at least in the case of IF and kinesthetic motor imagery exemplified in the referred study, interact mutually. Based on the cognitive-motor association, we previously focused on individual differences in attentional strategy and motor imagery ability during a visuomotor task. We found that the combination of an optimal strategy for directing attention and individual motor imagery ability can facilitate motor learning efficiency in the task^15^. Specifically, the EF strategy enhanced motor learning in participants with dominance of visual motor imagery, while the IF strategy enhanced motor learning in participants with dominance of kinesthetic motor imagery. These findings indicate that the EF strategy does not always lead to better motor performance in a healthy population.

However, to our knowledge, it remains unclear whether the combination of optimal attentional strategy and individual motor imagery ability improves motor performance in patients with motor disabilities after stroke. Previous studies demonstrated that the EF strategy induces greater hand velocities during reaching movements even in patients with mild to moderate paresis^16^,^17^. Although there is a speed-accuracy trade-off in motor control tasks^18^, most clinical studies focused mainly on movement velocity and not on both speed and accuracy for evaluating motor performance.

To address these gaps, we examined the effects of attentional strategy and motor imagery ability on performance of a simple motor control task in patients with acute stroke by measuring both movement errors and velocities. We hypothesized that motor performance under the EF would be superior to that under the IF in patients with visual-dominant motor imagery, while motor performance would be better under the IF in patients with kinematic-dominant motor imagery. Furthermore, we speculated that performance is better characterized by two measures, errors and hand movement velocity, than by movement velocity alone given the speed-accuracy trade-off.

## Methods

### Participants

**Figure 1:**
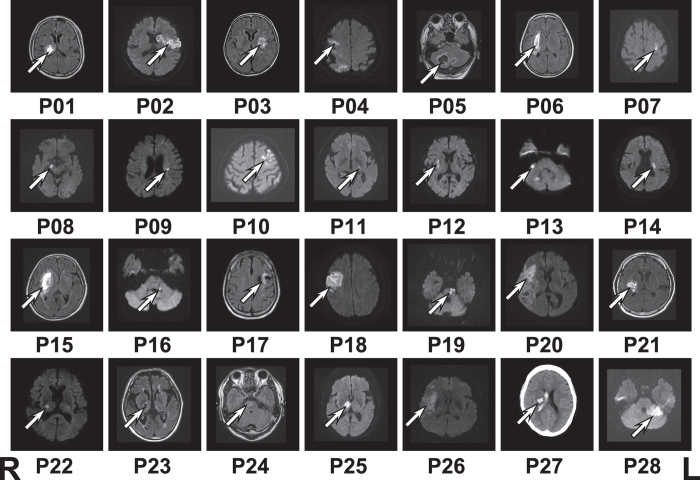
Lesion location of each patient. Patient number is according to modality dominance of motor imagery, P01 to P8 for kinesthetic dominance and P9 to P28 for visual dominance. Lesion locations were shown by fluid attenuated inversion recovery (FLAIR) MRI scans for hemorrhagic stroke (P01, P03, P05, P06, P15, P21, P23, and P24) and by diffusion-weighted image (DWI) MRI scans for ischemic stroke (P02, P04, P07−P14, P16, P18−P20, P22, P25, P26, and P28). Lesion location of P17 with ischemic stroke was shown by FLAIR MRI scan. Lesion location of P27 with hemorrhagic stroke was shown by CT scan. Left side of the figure represents the right side of the brain.

Twenty-eight patients with acute stroke were recruited from the Departments of Neurosurgery and Neurology of Jichi Medical University. At the first screening, we excluded patients with upper limb movement deficits unrelated to stroke, aphasia, dysarthria, visual field loss, and hemispatial neglect. The inclusion criterion was mild paresis of the tested upper limb (Manual muscle test grading ≥ 3). The exclusion criteria were severe sensory loss of the upper limb or Mini-Mental State Examination (MMSE)^19^ score less than 24. To evaluate the motor function after stroke, we applied the Fugl–Meyer Assessment of Motor Recovery (FMA). Twenty-eight healthy subjects with no neurological or skeletomotor dysfunction served as the control group. Each control participant was matched to a patient for age (within 2 years), sex, and hand dominance. Detailed participant information and lesion location of each patient are shown in Table 1 and Fig. 1, respectively. The Montreal Neurological Institute (MNI) coordinates of the local maxima within lesions were determined by the Statistical Parametric Mapping (SPM) anatomy toolbox^20^ and the Wake Forest University PickAtlas^21^ (details are shown in Supplementary Table S1). All participants provided written informed consent prior to the experiment, which was conducted in accordance with the Declaration of Helsinki and approved by the Institutional Review Board of Jichi Medical University.

### Protocol

#### Measuring ability of motor imagery

We first assessed motor imagery abilities of all participants using the short form of the Kinesthetic and Visual Imagery Questionnaire (KVIQ-10)^22^. The KVIQ-10 was specifically developed for assessing motor imagery ability in patients with restricted mobility. All movements were assessed with the participants in a sitting position. The questionnaire consists of 10 items, 5 each for evaluating kinesthetic and visual motor imagery, yielding separate kinesthetic and visual motor imagery subscale scores. The participants also evaluated the clarity of the image (visual) and the intensity of the sensation (kinesthetic) on a 5-point Likert scale. Visual motor imagery can be achieved from two perspectives, first person and third person^23^. Participants were explicitly instructed to imagine the movement from the first-person perspective. Movements included forward shoulder flexion, thumb−finger opposition, forward trunk flexion, hip abduction, and foot tapping. For each item, participants were asked to physically perform the movement as demonstrated by the experimenter and then to imagine the movement. To promote the first-person perspective, the experimenter sat beside the participant and demonstrated the action. After completing the mental task, participants were required to rate the vividness of imagery from 1 (no image, no sensation) to 5 (clear and intense image).

#### Apparatus for visuomotor task

Further, participants performed a simple visuomotor task that involved tracing a circular trajectory (radius of 7 cm) viewed on a monitor using a cursor controlled by a wireless computer mouse. During the visuomotor task, each participant was seated on a chair or wheelchair in front of a desk with a monitor. The distance between the participant’s eyes and the monitor was approximately 70 cm. All visual stimuli on the monitor were programmed in Matlab (MathWorks, Natick, MA) using Cogent Toolbox software (University College London, London, UK, http://www.vislab.ucl.ac.uk/cogent.php). The cursor position on the monitor (hand-cursor) was recorded using the toolbox with sampling at 60 Hz. The hand-cursor moved in synchrony with hand movements (Supplementary Fig. S1A).

#### Visuomotor circle-tracing task

This visuomotor task was chosen instead of the more complex motor learning task used in our previous study^15^ because most stroke patients had difficulty with the more complex task in preliminary trials. Participants in the stroke group were asked to hold the wireless computer mouse with their affected hand. For participants in the control group, we randomly selected the side to hold the wireless computer mouse. Eleven participants in the control group performed the visuomotor task with the right hand and the others with the left hand.

At the beginning of each trial, the participants were instructed to place their hand at the same location on the desk (i.e., in front of their body). Then, the hand-cursor was presented at the bottom of the desired circular trajectory. After the monitor displayed the desired circular trajectory and hand-cursor, the participant was required to trace the desired circular trajectory with the hand-cursor as accurately and quickly as possible by moving her/his hand in a circular pattern on the desk in the clockwise direction. We also instructed participants to control the mouse using only the upper limb and to keep her/his trunk stationary. Moreover, we supported the trunk as needed to minimize compensatory movements during the visuomotor task. For each trial, the participant was required to trace the trajectory for 15 seconds.

The visuomotor task was performed under three experimental conditions: no attentional instruction (NI), IF, and EF. For the NI condition, we did not provide any instructions regarding how to direct attention during trials. In the IF condition, participants were instructed to covertly direct attention to their hand movements. Namely, we instructed the participants to sense hand position and to move their hands in a circular trajectory on the desk. In the EF condition, participants were instructed to covertly direct attention to the cursor on the monitor. Namely, we instructed the participants to concentrate exclusively on the cursor movements on the monitor instead of directing attention on their hand movements. Thus, the only difference between the IF and EF conditions was the way of directing attention. We presented these attentional instructions for the IF/EF conditions before the beginning of each trial.

A previous study showed that the complexity of the instructions in a motor task can weaken difference between the IF and the EF conditions for novices^24^. Therefore, our current visuomotor task did not include a secondary task such as a reaction task as in our previous study^15^ to confirm the attentional direction (IF or EF) during the task. Rather, we asked the participants which way their attention was directed after completing all visuomotor tasks to confirm individual attentional strategy. All participants reported that attention to their hand was stronger under the IF condition than under the EF condition.

All participants were first asked to perform the visuomotor task under the NI condition (1st block), and then we assigned the IF or EF condition randomly for the 2nd and 3rd blocks (Supplementary Fig. S1B). Each block consisted of 10 trials; therefore, the participants completed 30 trials in total.

### Analysis

#### Ability of motor imagery

We assessed individual motor imagery ability by the total kinesthetic and visual motor imagery subscale scores on the KVIQ-10 questionnaire (maximum score of 25 for each modality). We classified the participants into kinesthetic- and visual-dominant subgroups according to the higher subscale score. However, when a participant showed equivalent total scores for kinesthetic and visual motor imagery, we asked them to choose which modality (kinesthetic or visual) was more vivid (forced choice).

#### Motor performance

Most previous motor control studies on patients with stroke focused mainly on movement speed during reaching tasks^16^,^17^. However, there is a speed-accuracy trade-off for motor performance^18^. Therefore, in addition to hand movement velocity, we also measured hand movement error as an index of motor accuracy to more precisely reveal the characteristics of motor performance in patients with acute stroke.

To express hand movement error, we first subtracted the radius of the specified trajectory from the distance between the fixation cross on the monitor and the cursor, frame by frame, and then averaged the values across a trial (i.e., 0–15 s). To express hand velocity, we divided the distance of hand movement in a trial by 15 seconds. To control for severity of paralysis in the stroke group, we normalized hand movement errors and velocities under both IF and EF conditions by dividing the respective means of each participant by those under the NI condition. Mean hand movement errors and velocities under both IF and EF conditions were also normalized to those under the NI condition in the control group.

### Statistical analysis

We compared both normalized hand movement errors and velocities in KVIQ-10 score subgroups (kinesthetic- or visual-dominant) using a mixed-design repeated measures analysis of variance (ANOVA) with Greenhouse–Geisser epsilon correction for nonsphericity.

A two-way ANOVA was applied to the KVIQ-10 score with participant group (stroke vs. control) as a between-subject factor and modality of motor imagery (kinesthetic vs. visual) as a within-subject factor. Three-way ANOVA was applied to both the normalized hand movement error and velocity. Participant group (stroke vs. control) and individual modality dominance (kinesthetic- vs. visual-dominant) were used as between-subject factors and attentional condition (IF vs. EF) as a within-subject factor. We also calculated the Pearson correlation coefficients to assess the relationships between the individual dominance of motor imagery and both indices of motor performance (normalized hand movement error and velocity). Furthermore, to test whether the normalized hand movement error and velocity obeyed speed-accuracy trade-off, we performed linear regression analysis. Both *F-* and *P-*values were then recalculated, and we considered statistical significance to be *p* < 0.05.

## Results

### Imagery scores

Both participant groups were better at visual motor imagery as measured by KVIQ-10 score [mean ± SEM; stroke group: 19.0 ± 0.68, control group: 19.6 ± 1.06] than at kinesthetic motor imagery [stroke group: 17.2 ± 0.78, control group: 18.7 ± 1.05]. Analysis of KVIQ-10 scores revealed a significant main effect of modality [*F*(1, 54) = 6.91, *p* = 0.011, *η*_*p*_^*2*^ = 0.11] but no main effect of group [*F*(1, 54) = 0.93, *p* = 0.34, *η*_*p*_^*2*^ = 0.02] or participant group × modality interaction [*F*(1, 54) = 0.40, *p* = 0.53, *η*_*p*_^*2*^ = 0.0073].

**Figure 2:**
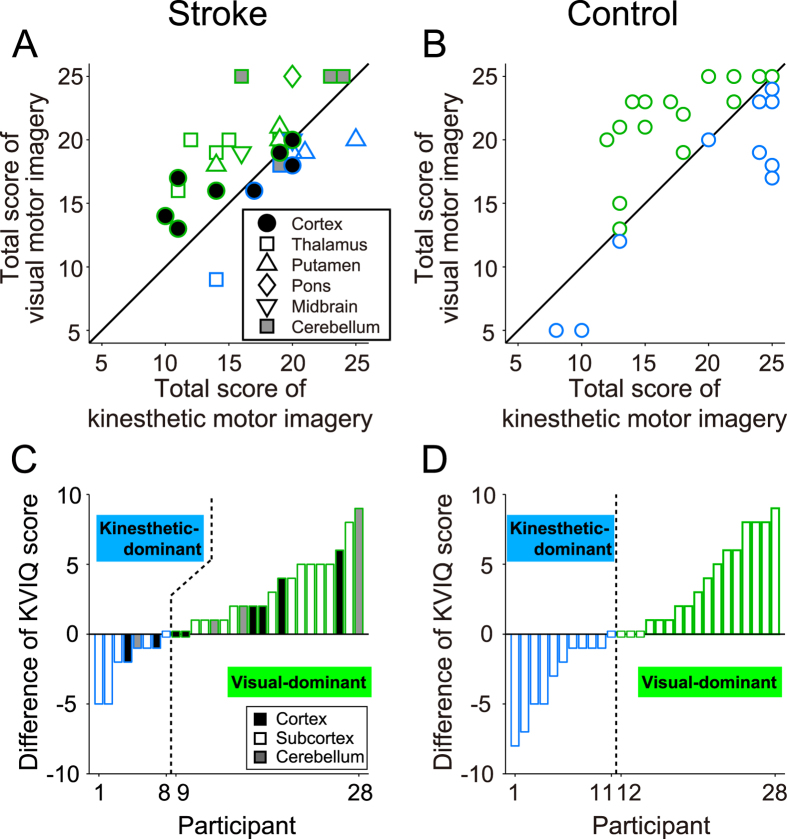
Individual differences in motor imagery ability and modality dominance. Scatter plots illustrate the distributions of motor imagery abilities in stroke (**A**) and control (**B**) groups. In stroke group, lesion locations are represented by shapes and color (white/gray/black). Data points below/above the diagonal line correspond to participants with higher kinesthetic/visual motor imagery ability (blue/green). The differential values between kinesthetic and visual motor imagery scores were sorted in ascending order in the stroke group (**C**) and the control group (**D**). Blue and green bars indicate participants who were kinesthetic- and visual-dominant, respectively.

Figure 2A and B illustrate the kinesthetic and visual score distributions in stroke and control groups. The diagonal lines in Fig. 2A and B indicate equal modality scores. The fractions of participants with higher visual scores than kinesthetic scores were 71.4% (20/28) in the stroke group and 60.7% (17/28) in the control group. Figure 2C and D show the differential modality scores on the KVIQ-10 (visual minus kinesthetic), where positive values indicate visual modality dominance and negative values kinesthetic modality dominance. Based on these differential values, we divided stroke patients into kinesthetic-dominant (n = 8; Participant Nos. 1−8) and visual-dominant (n = 20; Participant Nos. 9−28) subgroups and control participants into kinesthetic-dominant (n = 11; Participant Nos. 1−11) and visual-dominant (n = 17; Participant Nos. 12−28) subgroups.

### Normalized Hand Movement Errors and Velocities

**Figure 3:**
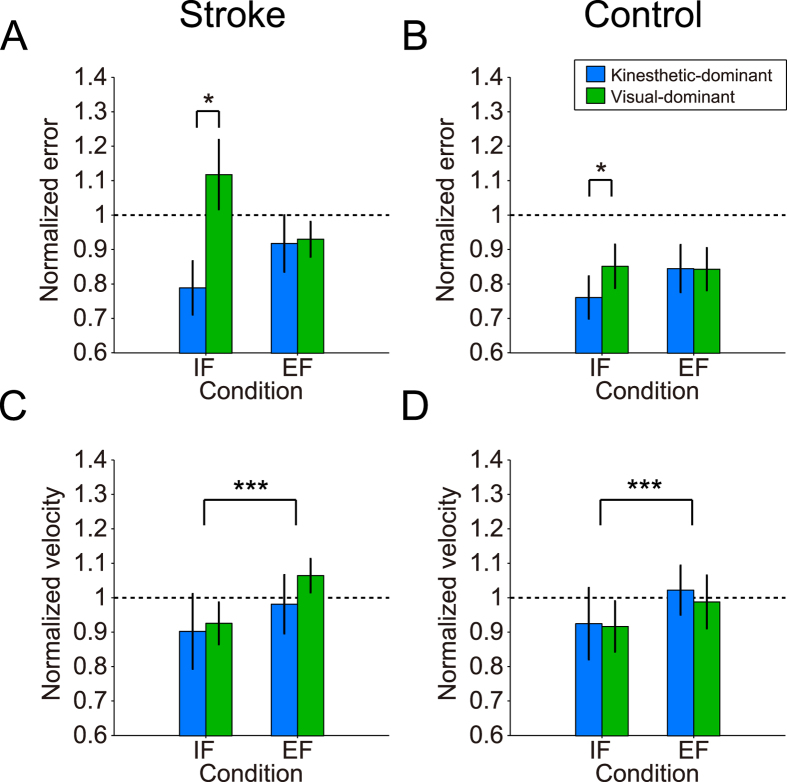
Normalized hand movement error (**A** and **B**) and velocity (**C** and **D**). Blue and green bars indicate participants with kinesthetic- and visual-dominant, respectively. Error bars denote standard errors. The participants who were visual-dominant showed significantly higher movement errors compared to those who were kinesthetic-dominant in the IF condition (*p = 0.044). On the other hand, regarding movement velocity, significantly faster velocity observed under the EF condition in both groups (***p = 0.0009).

Figure 3 shows the normalized motor performance in the stroke (Fig. 3A and C) and control (Fig. 3B and D) groups. Values higher than “1” denote higher movement error or faster hand velocity in the IF or EF condition compared to the NI condition. The influence of attentional strategy (IF vs. EF) on hand movement error strongly depended on individual modality dominance of motor imagery in the stroke group. For normalized hand movement error, we found significant interactions of dominance × condition [*F*(1, 52) = 6.38, *p* = 0.015, *η*_*p*_^*2*^ = 0.11], while other factors did not reach statistical significance [*F-values* < 2.55, *p-values* > 0.12]. Test results of the simple main effects for the significant interactions are shown in Supplementary Table S2. A post hoc analysis using the Bonferroni correction found that the participants with visual dominance made significantly more movement errors than those with kinesthetic dominance under the IF condition (*p* = 0.044; Fig. 3A and B).

Unlike normalized hand movement errors, normalized hand velocities did not depend on individual modality dominance of motor imagery. The main effect of condition was significant [*F*(1, 52) = 12.41, *p* = 0.0009, *η*_*p*_^*2*^ = 0.19], while effects of other factors did not reach statistical significance [*F-values* < 0.61, *p-values* > 0.44]. That is, in both groups, normalized hand velocity in the EF condition was significantly faster than in the IF condition (Fig. 3C and D).

### Relationship between imagery scores and motor performance

**Figure 4:**
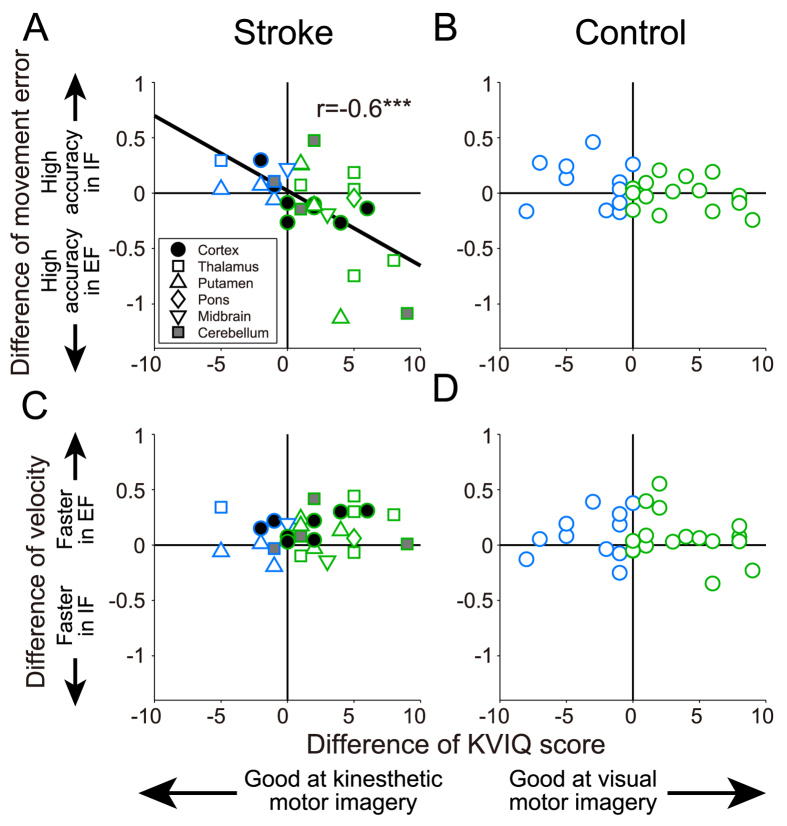
Correlations between individual dominant motor imagery modality and movement error (**A** and **B**), and velocity (**C** and **D**). In stroke group, movement errors were significantly correlated with individual motor imagery abilities (*r* = −0.6, ***p = 0.00073).

We explored the effect of individual motor imagery abilities on motor performance by analyzing the relationships between individual differential KVIQ-10 scores and both normalized hand movement errors (Fig. 4A and B) and normalized hand velocities (Fig. 4C and D). Positive values on the horizontal axes in Fig. 4 indicate that a participant was better at visual motor imagery than kinesthetic motor imagery, whereas negative values indicate better kinesthetic motor imagery than visual motor imagery. We also analyzed the differential values by subtracting the normalized hand movement errors and velocities under the IF condition from those under the EF condition. Positive values on the longitudinal axes in Fig. 4A and B indicate higher movement accuracy under the IF condition compared to the EF condition, and those in Fig. 4C and D indicate faster hand velocity under the EF condition compared to the IF condition. We found a significant negative correlation between movement accuracy and dominance of motor imagery in the stroke group (*r* = −0.6, *p* = 0.00073) but not the control group (*r* = −0.32, *p* = 0.10). These results suggest that patients with visual dominance showed more accurate movement under the EF condition, while patients with kinesthetic dominance showed more accurate movement under the IF condition. On the other hand, KVIQ-10 scores were not correlated significantly with normalized hand velocity in either participant group (*r* = 0.20, *p* = 0.31 in the stroke group; Fig. 4C. *r* = −0.12, *p* = 0.53 in the control group; Fig. 4D).

### Motor performance based on speed-accuracy trade-off

**Figure 5:**
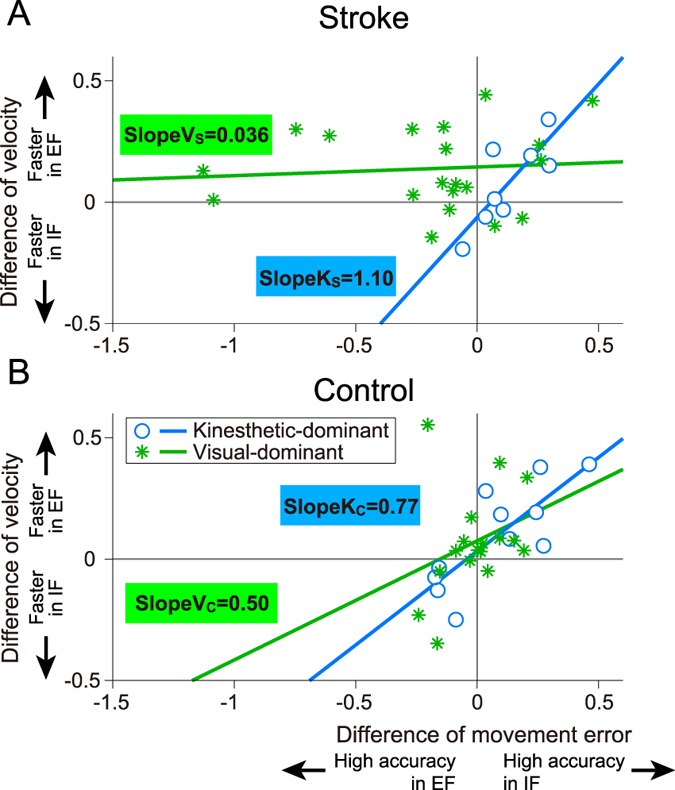
Relationships between speed (i.e., normalized hand velocity) and accuracy (i.e., normalized movement error) in stroke (**A**) and control (**B**) groups. Circles and asterisks indicate participants with kinesthetic- and visual-dominant imagery, respectively. The visual-dominant subgroup of stroke patients did not exhibit the typical speed-accuracy trade-off.

To explore the speed-accuracy trade-off, we analyzed the relationship between differential values of normalized movement error and velocity. Horizontal and longitudinal axes in Fig. 5 indicate differential values of the normalized hand movement error and velocity, respectively. Solid and dotted lines indicate the regression lines in kinesthetic- and visual-dominant subgroups, respectively. Linear regression analysis yielded a high *r* value for patients with kinesthetic dominance [*r* = 0.81, *F*(1, 6) = 11.06, *p* = 0.016]. That is, patients with kinesthetic dominance showed strong speed-accuracy trade-off (slope of the regression line: SlopeK_S_ = 1.10). Individuals who had higher accuracies showed slower movement speeds under the IF condition (circles on the first quadrant in Fig. 5A). Alternatively, the visual-dominant patient subgroup showed a greatly diminished speed-accuracy trade-off [*r* = 0.09, *F*(1, 8) = 0.14, *p* = 0.71, SlopeV_S_ = 0.036]. Over half of the patients with visual dominance showed improved speed and accuracy under the EF condition (asterisks on the second quadrant in Fig. 5A). In the control group, both kinesthetic- and visual-dominant subgroups showed positive slopes. In particular, the kinesthetic-dominant subgroup demonstrated strong speed-accuracy trade-off [Kinesthetic-dominant: *r* = 0.79, *F*(1, 9) = 14.72, *p* = 0.004, SlopeK_C_ = 0.77. Visual-dominant: *r* = 0.31, *F*(1, 15) = 1.56, *p* = 0.23, SlopeV_C_ = 0.50]. Fifteen of twenty-eight healthy individuals had higher accuracy and slower speed under IF (the first quadrant in Fig. 5B) and eight of twenty-eight healthy individuals had higher accuracy and slower speed under EF (the third quadrant in Fig. 5B).

## Discussion

In line with previous studies^16^,^17^, an increase was observed in hand movement velocity under the EF condition. However, in contrast with previous reports showing advantages of EF over IF during motor control tasks^1^,^2^ and in accordance with our initial hypothesis, we found that the combination of optimal attentional strategy and individual motor imagery ability improves movement accuracy in patients with motor disabilities after stroke. That is, movement accuracy under the IF condition was superior in patients with kinesthetic dominance than in patients with visual dominance. Furthermore, speed-accuracy trade-off in the stroke group strongly depended on individual modality dominance of motor imagery. Whereas the patients with kinesthetic dominance showed higher motor accuracy with lower velocity under the IF condition, patients with visual dominance showed both improved speed and accuracy under the EF condition.

Consistent with our previous findings in a healthy population^15^, we found a significant correlation between individual motor imagery ability and motor accuracy in the stroke group. However, other indices that characterize motor performance, such as velocity and accuracy of hand movements, were somewhat inconsistent with previous studies^16^,^17^. The violation of speed-accuracy trade-off found in patients with visual dominance under the EF condition suggests that motor control can be achieved more efficiently. Indeed, patients showing improvements in both speed and accuracy demonstrated more successful motor learning compared to participants susceptible to the speed-accuracy trade-off^25^,^26^. Our findings clearly showed that EF was the optimal strategy for patients with visual dominance. On the other hand, patients with kinesthetic dominance exhibited a speed-accuracy trade-off, suggesting that they could perform accurate arm movements by reducing motor speed. Thus, considering the relationship between skilled motor training and motor recovery^27^, IF may be an optimal strategy to enhance rehabilitation benefits for patients with kinesthetic dominance.

Externally focused attention has been considered the more effective strategy for motor control tasks based on the constrained-action hypothesis, which presumes that conscious monitoring and control of body movement (IF strategy) interferes with automatic motor control processes whereas this interference can be weakened by the EF strategy^6^. Further, predictive eye movements towards the goal during goal-directed motor tasks^28^ would support EF as the spontaneous (natural) attentional strategy. However, contrary to these traditional views, we found that IF might be an effective strategy for stroke patients. Hand movement error significantly correlated with hand velocity, and this speed-accuracy trade-off suggests that IF is the optimal attentional strategy for stroke patients with kinesthetic dominance, allowing them to control hand movements with higher accuracy. As we have previously demonstrated, it is likely that inconsistent findings across studies are partly due to the individual history of motor learning or sports training, which can lead to a preferred sensory processing modality^15^.

A previous study also noted that therapists generally request internally focused attention, which would hinder learning and retention of rehabilitation^29^. Further, a recent study reported that patients with stroke may have an inherent preference for the IF strategy^30^. We have clearly demonstrated that motor performance depending on attentional strategy is not always subject to speed-accuracy trade-off. Therefore, the combination of the individual dominant modality of motor imagery and the more suitable individual attentional strategy based on dominant motor imagery ability enhances movement accuracy. These findings imply that assessing the individual’s optimal attentional strategy is necessary prior to rehabilitation. However, note that several patients showed weak modality dominance (10 patients had a difference of KVIQ score of ±1; Participant Nos. 5−14 in stroke group). This may be partly because dominance of motor imagery tends to abate with age^31^. Therefore, both the questionnaire and a motor task would be effective as a pre-assessment tool. Further, our results suggest that the tailor-made instructions based on the pre-assessment procedures would maximize individual benefits of rehabilitation for functional recovery.

Although the stroke group included patients with widely distributed lesions (in cortex, thalamus, putamen, pons, midbrain, and cerebellum), we did not find lesion-specific modulation of motor performance by attentional strategy or individual motor imagery ability. It is possible that the brain regions critical for both, such as the inferior parietal lobule, were intact in all patients^14^. Therefore, it is likely that we can apply our current experimental paradigm to characterize the optimal individual attentional strategy for patients free from injuries in these regions. However, low number of patients in this study did not allow determining whether a correlation exists between lesion site and optimal attentional strategy or motor imagery outcomes. Further studies with larger patient populations are needed to clarify this point.

Contrary to the stroke group and our previous study of healthy participants^15^, we did not find a significant correlation between hand movement errors and individual motor imagery ability in the control group. One possible reason for this discrepancy is task difficulty. When the motor task is too easy, these attentional effects are not expected^32^,^33^,^34^. As we found that our previous task^15^ was very difficult for stroke patients in our preliminary tests, we applied a simpler task. However, this task was likely insufficiently difficult in the healthy population to induce significant attentional effects on motor performance.

The limitation of this study was that our current task was rather simple compared to those used in clinical practice, so it is also necessary to verify whether our current results hold true for complex motor tasks more reflective of daily activities. Although current findings could guide the development of tailor-made pre-assessment procedures and rehabilitation programs optimized for individual cognitive abilities, further studies using retention tests are warranted to explore the applicability of individual suitable attentional strategy on motor recovery in mid- and long-term rehabilitation programs.

## Additional Information

**How to cite this article**: Sakurada, T. *et al*. Improved motor performance in patients with acute stroke using the optimal individual attentional strategy. *Sci. Rep.* **7**, 40592; doi: 10.1038/srep40592 (2017).

**Publisher's note:** Springer Nature remains neutral with regard to jurisdictional claims in published maps and institutional affiliations.

## Supplementary Material

Supplementary Information

## Figures and Tables

**Table 1:** Participant information.

Variable (Values are mean ± SD)	Stroke group	Control group
n	28	28
Age (years)	64.9 ± 10.6	65.1 ± 10.2 (*p* = 0.92)
Gender	10 F/18 M	10 F/18 M
Handedness	28 R/0 L	28 R/0 L
Time since stroke (days)	11 ± 7.1	N/A
Affected side	11 R/17 L	N/A
Stroke lesion		
Cortex	8	N/A
Subcortex	16	
Cerebellum	4	
Stroke type		
Hemorrhagic	9	N/A
Ischemic	19	
MMSE (/30)	27.1 ± 1.3	28.6 ± 1.4 (*p* = 0.002)
Fugl–Meyer score (/66)	58.0 ± 5.4	N/A
