# Supplementary material for: Improved motor performance in patients with acute stroke using the optimal individual attentional strategy
Source: Sci Rep. 2017 Jan 17;7:40592. doi: 10.1038/srep40592 (PMC5240116; doi:10.1038/srep40592)
Supplement: Supplementary Information [file srep40592-s1.doc]

**Supplementary Information**

**Title**

Improved motor performance in patients with acute stroke using the optimal individual attentional strategy

**Authors**

Takeshi Sakurada, Takeshi Nakajima, Mitsuya Morita, Masahiro Hirai and Eiju Watanabe.

**Supplementary Figure S1: Experimental setup**


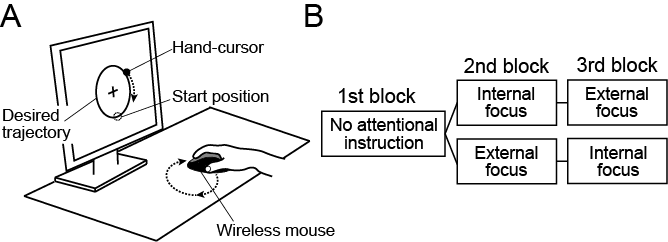


**A.** Participants were required to trace a desired trajectory (circle) in the clockwise direction on a computer monitor by moving a wireless computer mouse. The location of the hand-cursor on the monitor was defined by the location of the mouse. **B.** Experimental procedure. In the 1st block, all participants performed the visuomotor tracing task without any attentional instruction (NI condition). In the 2nd block, the participants were required to covertly direct their attention to either their hand (IF condition) or to the hand-cursor (EF condition) as determined randomly. In the 3^rd^ block, participants were instructed to use the other attentional strategy for the same task. In all conditions, participants were required to fixate on the center of the monitor.

**Supplementary Table S1: MNI coordinates of local maxima within** lesions

| Patient number | Localization | MNI coordinates | | |
| --- | --- | --- | --- | --- |
|  |  | *x* | *y* | *z* |
| P01 | R Thalamus | 16.2 | -20.4 | 19.0 |
| P02 | L Putamen | -10.6 | 8.7 | -5.7 |
| P03 | L Putamen | -29.1 | -12.5 | 7.3 |
| P04 | R Precentral Gyrus | 39.3 | -9.7 | 48.9 |
| P05 | R Cerebellum | 10.6 | -65.9 | -38.0 |
| P06 | R Putamen | 30.0 | -0.5 | 0.8 |
| P07 | R Precentral Gyrus | 48.5 | -3.2 | 40.6 |
| P08 | R Midbrain | 15.2 | -16.2 | -7.5 |
| P09 | L Frontal Lobe | -25.4 | -14.3 | 28.6 |
| P10 | L Middle Frontal Gyrus | -29.1 | 2.3 | 54.4 |
| P11 | L Thalamus | -18.9 | -19.8 | 3.6 |
| P12 | R Putamen | 30.9 | -2.3 | 13.8 |
| P13 | R Cerebellum | 30.9 | -31.8 | -37.1 |
| P14 | L Putamen | -25.4 | -9.7 | 11.9 |
| P15 | R Putamen | 29.1 | -1.4 | 2.7 |
| P16 | L Cerebellum | -8.8 | -47.5 | -34.3 |
| P17 | L Precentral Gyrus | -34.6 | 5.0 | 48.9 |
| P18 | R Precentral Gyrus | 51.3 | 0.4 | 30.4 |
| P19 | L Midbrain | -9.7 | -24.5 | -9.4 |
| P20 | R Inferior Frontal Gyrus | 48.5 | 18.9 | 20.2 |
| P21 | R Putamen | 27.2 | -9.7 | 6.4 |
| P22 | R Thalamus | 20.8 | -16.2 | 11.0 |
| P23 | R Thalamus | 13.4 | -13.4 | 14.7 |
| P24 | R Pons | 7.8 | -22.6 | -31.5 |
| P25 | R Thalamus | 14.3 | -14.3 | 7.3 |
| P26 | R Inferior Frontal Gyrus | 47.6 | 5.0 | 17.5 |
| P27 | R Thalamus | 20.8 | -21.7 | 14.7 |
| P28 | L Cerebellum | -24.5 | -54.0 | -16.8 |

**Supplementary Table S2: Simple effects of modality dominance × condition interaction on normalized hand movement error**

| Effect | *F-value* | *p-value* |
| --- | --- | --- |
| A(b1) | *F*(1, 104) = 6.56 | 0.012* |
| A(b2) | *F*(1, 104) = 0.004 | 0.95 |
| B(a1) | *F*(1, 52) = 3.46 | 0.069 |
| B(a2) | *F*(1, 52) = 2.94 | 0.093 |

Source A: Modality dominance (a1: kinesthetic, a2: visual), Source B: Condition (b1: IF, b2: EF).

**p* < 0.05
